# Priming Children’s Use of Intentions in Moral Judgement with Metacognitive Training

**DOI:** 10.3389/fpsyg.2016.00190

**Published:** 2016-03-18

**Authors:** Katarina Gvozdic, Sylvain Moutier, Emmanuel Dupoux, Marine Buon

**Affiliations:** ^1^Laboratoire Paragraphe, Saint-Denis University Saint Denis, France; ^2^Laboratoire de Psychopathologie et Processus de Santé, Paris Descartes UniversityParis, France; ^3^Laboratoire de Sciences Cognitives et de Psycholinguistique, Department of Cognitive Studies, Ecole Normale SupérieureParis, France; ^4^Institut Jean Nicod, Department of Cognitive Studies, Ecole Normale SupérieureParis, France

**Keywords:** moral development, theory of mind capacities, inhibitory control resources, dual-processes, metacognition

## Abstract

Typically, adults give a primary role to the agent’s intention to harm when performing a moral judgment of accidental harm. By contrast, children often focus on outcomes, underestimating the actor’s mental states when judging someone for his action, and rely on what we suppose to be intuitive and emotional processes. The present study explored the processes involved in the development of the capacity to integrate agents’ intentions into their moral judgment of accidental harm in 5 to 8-year-old children. This was done by the use of different metacognitive trainings reinforcing different abilities involved in moral judgments (mentalising abilities, executive abilities, or no reinforcement), similar to a paradigm previously used in the field of deductive logic. Children’s moral judgments were gathered before and after the training with non-verbal cartoons depicting agents whose actions differed only based on their causal role or their intention to harm. We demonstrated that a metacognitive training could induce an important shift in children’s moral abilities, showing that only children who were explicitly instructed to “not focus too much” on the consequences of accidental harm, preferentially weighted the agents’ intentions in their moral judgments. Our findings confirm that children between the ages of 5 and 8 are sensitive to the intention of agents, however, at that age, this ability is insufficient in order to give a “mature” moral judgment. Our experiment is the first that suggests the critical role of inhibitory resources in processing accidental harm.

## Introduction

**FIGURE 1:**
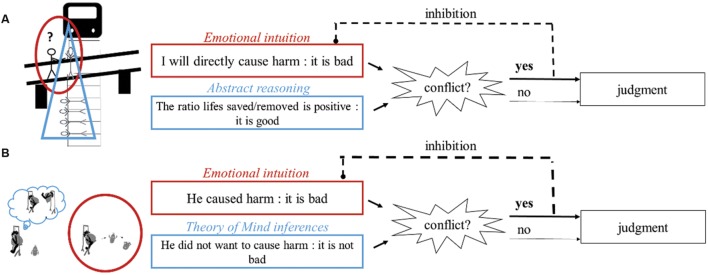
**Schematic illustration of Greene’s dual processes model (Greene, 2009) applied to **(A)** the Footbridge dilemma and **(B)** accidental harm.** In the Footbridge dilemma, a runaway trolley is headed for five people. You (with the question mark) are standing next to a man on a footbridge spanning the tracks where the only way to save the five people is to push this man off the footbridge and into the path of the trolley. When participants have to decide whether they would push the man, they undergo a cognitive conflict. On one side, the system responsible for the evaluation of the action to be performed generates an automatic emotional aversion that leads the participants to condemn the action. One the other side, the system responsible for the rational evaluation of the action’s consequences (the action will save more lives than it will cause deaths) leads them to consider this action as permissible. To solve the conflict rationally, people need to deploy inhibitory control to override their negative intuition arising from the evaluation of the action to be performed. In the case of accidental harm, there is an individual that caused harm without the intention to do so (e.g., he just wants to swing and strucks another individual because he did not see him arriving). According to our hypothesis, on one side the system responsible for the evaluation of agent’s causal role generates an automatic emotional aversion toward the person responsible for the suffering of a victim. On another side, the system responsible for the evaluation of the agent’ intention leads people to evaluate positively the agent.

Currently, multiple factors are considered to influence our moral judgment competencies (Cushman et al., 2010; Young and Dungan, 2011). These factors include emotional and intuitive processes (Greene et al., 2001), abstract reasoning abilities (Greene et al., 2004), theory of mind (ToM) capacities (Young et al., 2007), and executive control resources (Greene et al., 2004; Moore et al., 2008; Buon et al., 2013b). Even though their exact contribution, interaction and/or potential competition in adults’ moral judgments are not well established and vary across different theoretical points of view, most scholars now agree that moral judgments depend on both intuitive and controlled processes, and that moral competences rest upon the ability to deal with and integrate conflicting moral and social considerations (Smetana and Killen, 2008; Young and Dungan, 2011; Buon et al., 2015). For instance, according to Greene’s (2009) dual process model, based on the now famous Footbridge dilemma (see **Figure 1A**), emotional/intuitive systems tend to dominate people’s judgments in situations of high conflict, unless they are able to deploy important inhibitory control resources to engage in and make use of more rational considerations (for a review, see Cushman et al., 2010).

This paper aims to explore the validity of a dual process architecture in order to understand an important landmark in children’s moral *development*: the ability to generate intent-based moral judgments. Typically, adults tend to blame individuals for their intent to cause harm, even if they did not manage to fulfill their malevolent intent (i.e., in case of attempted harm). Conversely, we tend to judge more leniently someone who caused harm without the intention to do so (i.e., accidental harm; Piaget, 1965/1932; Cushman, 2008). This ability to prioritize information about individuals’ intentions when producing a moral judgment is thus a critical feature of adults’ morality; however, it is not until late in development that children are able to do the same. As Piaget’s (1965/1932) work demonstrated, children until the age of 8/9 years tend to focus on the outcomes when making moral judgments about someone’s act, while ignoring the protagonist’s intention. For instance he showed that young children considered that a boy breaking eight cups accidentally is naughtier compared to a boy who tried to take some jam while his mother was away and only broke one cup. Later studies, using more various and child-friendly methodologies, have refined the initial developmental pattern proposed by Piaget. In particular, they have reported that under optimized conditions (e.g., stimuli including an explicit and salient description of the protagonist’s mental states; showing stimuli by pairs of stories differing on only one criteria, i.e., intentions or outcomes), preschoolers between the age of 3 and 5 could be able to judge someone committing intentional harm as naughtier than someone committing accidental harm (Nelson, 1980; Baird and Astington, 2004; Nobes et al., 2009; Cushman et al., 2013, but see Imamoğlu, 1975; Zelazo et al., 1996; Helwig et al., 2001). Yet, Piaget’s main claim has never been completely challenged since most of those post-Piagetian studies have also reported an increase in the use of information about intentions and a decrease in the use of outcomes between the ages of 3–5 and 7–9 (Nelson-Le Gall, 1985; Shultz et al., 1988; Baird and Astington, 2004; Nobes et al., 2009; Cushman et al., 2013). Put simply, even though preschoolers show some sensitivity to a protagonist’s intent when making moral judgments, their ability to *prioritize* information about intentions undergoes a protracted development. Interestingly, in one recent study, the authors demonstrated that this late emergence mainly regards cases of accidental harm and not attempted harm (Cushman et al., 2013). That is, whereas 5-year-old children are as likely as older children to blame individuals who commit attempted harm, it is not until 8 years of age that children are able to judge accidental harm leniently. What could be the cognitive mechanisms underlying this late moral development?

To date, most studies that have investigated the mechanisms underlying the ability to generate intent-based moral judgment have focused on the critical role of ToM, the ability to represent and infer individuals’ mental states and to use them to explain and predict others’ behaviors (Premack and Woodruff, 1978). Especially, adult studies have reported evidence in favor of a relationship between ToM and moral judgment (Knobe, 2005; Moran et al., 2011; Buon et al., 2013a), as well as neuroscience research, which demonstrated an important role of brain regions responsible for mental states attribution in judgments of blame ascription (Young et al., 2007, 2010; Young and Saxe, 2009). From a developmental perspective, several studies have reported a significant relationship between children’s ToM competencies (assessed by their ability to pass the false belief task^1^) and their ability to generate intent-based moral judgment (Baird and Astington, 2004; Killen et al., 2011; Fu et al., 2014). For instance, Killen et al. (2011) have demonstrated that children who passed the false belief task were more likely, than their peers who did not, to assess the intention of someone accidentally harming someone else as nice, and to evaluate her action leniently. Even though this does suggest that ToM is indeed critical for judging cases of accidental harm, it should be noted that the ability to pass the FBT has been shown to also involve general purpose skills, including executive functions resources (Carlson et al., 2004; Apperly et al., 2006), therefore limiting a clear-cut interpretation of those findings.

Another cognitive tool that may be highly useful for children (and adults) to generate intent-based moral judgment is inhibitory control- the ability to tune out stimuli that are irrelevant to the task or process at hand or to the mind’s current state (Macleod, 2007). In addition to its potential involvement in ToM reasoning (Carlson et al., 2004; Apperly et al., 2006), several authors have proposed that processing accidental harm may be underlined by the same type of cognitive architecture as the one required to process the Footbridge dilemma described above (Young et al., 2007; Buon et al., 2013b; Cushman, 2014, see also **Figure 1B**). In short, when faced with accidental harm, individuals first have an (negative) intuitive and emotional reaction toward the protagonist who carried out this action based on the perception of the harm he caused. This negative appraisal of the protagonist would then enter in conflict with a more positive evaluation built through ToM inferences about the agents’ innocent intent. Finally, in line with Greene’s proposal, in order for this conflict to be solved in favor of non-emotional and more rational considerations (i.e., the protagonist’s innocent intentions), individuals need to override their initial negative intuition with the help of their inhibitory control. If this proposition was correct, 5 to 7-year-olds selective difficulty to judge accidental harm would be mainly explained by their inhibitory control limitations. Evidence in favor of this hypothesis is quite numerous in adults studies. Indeed, when judging accidental harm, adults have been shown to be in a situation of cognitive conflict (Young et al., 2007) and recent findings assert that whereas an adult’s evaluation of the protagonist’s causal role relies upon intuitive and emotional mechanisms when faced with accidental harm, the integration of its neutral intent in their moral judgment would require costly cognitive mechanisms (Young et al., 2012; Buon et al., 2013b). Yet, when it comes to the *development* of the ability to generate intent-based moral judgments, though the importance of inhibitory control capacities has been proposed by several authors (Zelazo et al., 1996; Richardson et al., 2012; Cushman et al., 2013), no study has yet tested this directly. Nevertheless indirect evidence may argue in favor of this hypothesis. In particular, several studies suggest that preschoolers are much more likely to generate intent-based moral judgments when the consequences are absent, or at least kept constant in the stimuli compared by participants (e.g., see Baird and Astington, 2004). This experimental specificity may in fact attenuate the conflict that occurs between the information about the protagonist’s intent and the consequences of his/her actions and therefore reduce the need to deploy inhibitory skills, making the task easier for children.

Investigating the critical role of inhibitory control in making mature intent based moral judgments is not only in line with recent modeling of adults’ moral competencies (Greene, 2009), but also goes well with recent models of children’s social and cognitive development. First, it fits the process-based account recently advanced by the social domain theory (Richardson et al., 2012), according to which two competing systems could be at work when making socio-moral decisions: an unreflective system that tends to focus on the outcomes of a harmful action, and a (reflective) system that tends to focus on the protagonist’s intention. Accordingly, when faced with accidental action, young children have difficulties to override the experience system, which likely encourages a focus on the outcomes (e.g., if the outcome is bad then the intentions and actions are bad) whereas older children would be more and more able to override the experiential system by the representational one, with the help of their executive development. Secondly, and more broadly, the idea that inhibition may play a key role in the emergence of intent-based moral judgment allows to extend some of the recent neo-Piagetian views of cognitive development to moral competences (Houdé and Borst, 2015). According to such a view, inhibitory control is essential to override the automatic/heuristic responses that adults and children rely on heavily and activate more costly algorithms (i.e., slow, analytical, and cognitively costly strategies that always provide the correct solution). To date, using specific methodologies (see for a review, Houdé and Borst, 2014), several studies have supported the role of inhibition in the development of logical and reasoning abilities. For instance, using a carefully constructed metacognitive training^2^, Moutier (2000), Moutier et al. (2002), and Moutier and Houde (2003), have shown that the difficulties of school-age children (but also adults) to solve a simple deductive reasoning task are not due to a lack of logic but instead to an inability to inhibit a perceptual bias triggered by the task instructions. In particular, they demonstrated that whereas children did not improve their ability to solve the conditional rule falsification task^3^ when they were explicitly taught to use a logical truth table (‘logical component’ training), their performance clearly improved when logical rules were paired with explicit alerts about the need to inhibit a perceptual bias (‘logical + inhibitory component’ training), confirming that overriding an automatic response plays a central role in accessing a more elaborate analysis of the situation.

The aim of the present study is to investigate whether the children’s capacity to generate intent-based moral judgment relies on the need to inhibit their intuitive response to harmful outcomes. As stressed above, we hypothesized that children aged 5 to 7/8 years do not produce a “mature” (intent-based) moral judgment due to difficulties in inhibiting their intuitive/emotional reaction that emerges when faced with a harmful outcome. In order to test this hypothesis, building onto a metacognitive training, such as the one described above (Moutier, 2000; Moutier et al., 2002; Moutier and Houde, 2003), made to help the participants engage in a reflective process about their decisions. The paradigm we developed aimed at instructing children to take into account the protagonist’s mental state (mentalising component), to which we added (or did not add) executive alarms (inhibitory component). The executive alarms were designed to warn participants about the tendency to base our judgment on the automatic reaction toward the victims’ suffering. Importantly, those type of alarms do not aim to improve children’s general inhibitory control skills but to increase their ability to strategically deploy inhibitory control to solve the task. In total, we created three metacognitive trainings: the mentalising reinforcement (MR) training that included the mentalising component only, the metacognitive training with mentalizing and inhibitory reinforcement (MIR) that included both the mentalising and executive component and a control reinforcement, without particular reinforcement, used as a control for any test–retest effect.

To investigate the impact of our different metacognitive trainings on 5 to 8-year-old children’s moral abilities, moral judgments were measured before and after the training, using an experimental paradigm adapted from Buon et al. (2013b). We gathered children’s judgments about protagonists involved in a simple coincidence (a victim suffers but the protagonist does not cause harm and does not have a harmful intention), an accidental harm (a protagonist causes harm while having a harmless intention), and an aggression (a protagonist causes harm while having a harmful intention). The situations were represented using pairs of non-verbal cartoons, each pair contrasting protagonists based either on the protagonists’ causal roles (causal contrast: coincidence versus accident) or the protagonists’ intentions to harm (intentional contrast: accident versus aggression). Children were then asked to evaluate each protagonist individually and, by comparing protagonists presented in a given pair, we obtained an index of each child’s moral sensitivity to protagonists’ causal role and protagonists’ intention to harm. In short, all children first underwent a (pre-test) moral task designed to assess their initial moral sensitivity to protagonists’ causal roles and their intention to harm, and thus their ability to give more weight to the agent’s’ intentions in their moral judgment. Then, they were randomly assigned to one of the three metacognitive training described above (control, mentalising, or inhibitory reinforcement). Finally, in order to examine the impact of our different metacognitive trainings on children’s moral judgment, participants passed a post-test, in which they underwent the same moral task as in the pre-test. The post-test was identical to the pre-test in order to be able to attribute any observed effect solely to the training and avoid any possible confounding factors^4^.

Note also that in order to establish the developmental validity of our paradigm, a group of 16 healthy adults were administered in the pre-test (average age 26.12, nine women, see **Supplementary Material S01**).

The predictions were as follows: firstly, we expected that during the pre-test, children’s moral judgments would be equally sensitive to the protagonist’s causal role and intention to harm, whereas adults’ moral judgments would be *mostly* sensitive to protagonist’s intentions. This would reflect the developmental pattern introduced above, according to which, even though children start to integrate protagonists’ intentions from an early age, they are not able, contrary to adults, to prioritize this information in their moral judgment before 8/9 years of age. Secondly, we expected that only the training including an executive warning about the need to inhibit a preponderant emotional response (Metacognitive training with MIR) will have an effect on children’s moral judgments, allowing them to generate an intent-based moral judgment. This would demonstrate that 5 to 8-year-old school-age children’s difficulty to generate intent-based moral judgment do not come from a lack of sensitivity to protagonists’ intentions but from an inability to inhibit their intuitive reactions to harmful outcomes.

## Materials and Methods

### Population

Native French speaking children between the ages of 5 and 8 years were recruited at the science museum in Paris “La cite des sciences,” accompanied by their parents. A total of 102 children participated, but 30 children were removed from the analysis (one was below the age range, 29 children unsuccessfully completed the metacognitive training protocol: CR training, *n* = 2; MR training, *n* = 12, MIR training, *n* = 15). This drop rate may appear abnormally high compared to other metacognitive trainings that have been previously done in the literature. However, it should be noted that, in contrast to previous experiments of this kind that repeated the trainings as many times as necessary (Moutier, 2000; Moutier et al., 2002; Moutier and Houde, 2003), we decided to repeat the resume of the metacognitive training only once (see procedure below) which may justify why we had to remove a substantial number of children. In total, 72 children successfully completed the metacognitive training protocol. In each group, there was a total of 24 children. The mean age of the children was 6.97 (CR group: 6.72 years; MR group: 7.01; MIR group: 7.17 years^5^). Of the 72 participants, 42 were girls.

Sixteen healthy adults recruited at the Ecole Normale Supérieure (Paris, France) also completed the pre-test of our experiment (mean age = 26.12 years). All adults were volunteers who agreed to participate in the study.

### Stimuli

#### Videos

For the pre- and post-test sessions of the experiment we used videos that have previously been validated and used in a study by Buon et al. (2013b), as well as new videos that has been validated in a pilot study using 16 adult participants (see **Supplementary Material S01** for results of the pilot study). The videos were constructed in Adobe Flash 8.0. and lasted 10 s, with either Mr. Blue or Mr. Green as the protagonist, swinging near a road (on a swing or a rope by the road scenario), or Mr. Gray and Mr. Yellow, (swinging on a trapezoid or on a bar in a park scenario). They all had Mr. Red as the victim.

**FIGURE 2:**
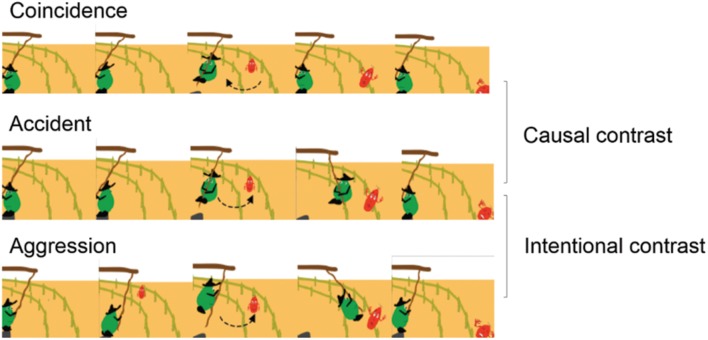
**Synopsis of the three conditions used in the pre- and post-test sessions of the experiment.** In the Coincidence scenario, the protagonist does not hit Mr. Red who falls on his own. In the Accident scenario, the protagonist hits Mr. Red without knowing that Mr. Red was on the road. In the Aggression scenario, the protagonist intentionally hits Mr. Red.

For each protagonist, there were three different conditions (see **Figure 2**). In the aggression conditions, the protagonist faces the road, swings just once and stops. Then, he looks at the road as the victim (Mr. Red) is approaching and starts swinging again and intentionally hits Mr. Red when he is standing right in front of him. The aggression condition is therefore characterized by a negative intention and a negative causal role of the agent. In the accidental conditions, the protagonist is facing away from the road. He looks at the road while there is nobody (for the same duration as in the aggression conditions), and starts swinging. While the protagonist is swinging, Mr. Red, who is walking by, is accidentally hit by the second swinging action In this condition, the agent therefore has a neutral intention, since he showed a sign of not wanting to cause harm, but has, however, a negative causal role. In the coincidental conditions, the protagonist’s movements are identical to those displayed in the accidental conditions except that they are shifted in time (0.5 s), so that he stops swinging before the victim stumbles by his own doing, characterizing the condition with a neutral/positive intention and a neutral causal role of the agent since the negative outcome cannot be ascribed to the agent’s actions. In sum, the three types of conditions contained the same actions, with the only difference being their relative timing and the orientation of the protagonists (facing toward vs. away from the road).

Conditions were paired so as to form different contrast measures (differing in only one aspect): the causal contrast – comparison of the accident and the coincidence scenarios – yielding a measure of the influence of the protagonist’s causal role in moral/social evaluations; or the intentional contrast – comparing the accident and the aggression scenarios – yielding a measure of the influence of intention ascription in moral/social evaluations.

#### Training Stimuli

**FIGURE 3:**
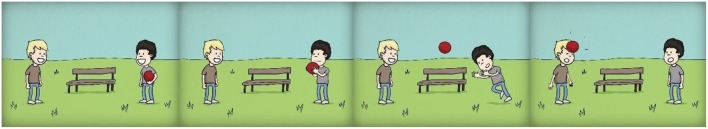
**Baseline images used during the metacognitive training session to illustrate the story plot-line presented to the child.** In the story, two boys are playing together. One of the boys is throwing the ball but accidentally trips and the ball falls on the other boy’s head injuring the other boy.

We built three types of metacognitive trainings (**Table 1**) to which the children were randomly assigned, aiming at different objectives: (1) a control reinforcement (CR) – without instructions to focus specifically on one component of the situation; (2) a MR – aimed at reinforcing the children’s focus on mental states; (3) MIR – aimed at warning the children about the need to inhibit their automatic reaction toward the victim’s suffering, alongside with reinforcing the child’s capacities to focus on mental states. The three metacognitive trainings all used the same baseline images, constructed for this experiment, illustrating two boys playing with a ball (see **Figure 3**). In the story, two boys are playing together. One of the boys is throwing the ball but accidentally trips and the ball lands on the other boy’s head injuring the other boy. For all three trainings, we first started by presenting the story plot-line to the child. The child was then asked to judge whether the boy who threw the ball was nice or mean.

**Table 1:** Statements told to the child at the introduction (before the black bar) and the summarize of the training (after the black bar), as a function of the type of training.

CR	MR	MIR
(…)	I suggest we look at all the different elements of the story in order to better understand, how we can give these different responses.	
		Indeed, there is a trap here, which is to only look at certain elements of the story, for instance to only look at the blond boy getting hurt at the end of the story. The risk would be to forget other elements of the story that might help us respond to the question

	So, in order to respond to the question it is important	

		to not to fall in to this trap, that is to say not to look at just one single element of the story and

	to look at all of the elements of the story, including what the brown haired boy wanted to do in the beginning of the story	

Let’s look at the different responses: if we only look at the blond boy being hurt at the end of the story after the dark haired boy threw the ball, we could have the need to say that the dark haired boy is mean.		

So, if we look at the fact that the dark haired boy did not actually want to hurt the blond boy, we can give a different response to the question of whether the dark haired boy is mean or nice, and we can say that he is in fact nice.		

	So, to summarize, when we see someone getting hurt, in order to say if the person who hurt him is nice or mean, we should try and look at all the elements of the story. So here we need:	

		First not to fall into the trap and the gray cage helps us do this. We must not look at only the end of the story when the blond boy gets hurt, otherwise we could automatically respond saying that the dark haired boy is bad and we risk forgetting other elements from the beginning of the story which are important and can lead us want to a different response. Second, once we have avoided the trap, we can look at different elements of the story since it is also important.

	To ask ourselves what the dark haired boy actually wanted to do at the beginning of the story, which the yellow circle helps us remember. Since the dark haired boy tripped and since the ball flew in an unwanted direction, we can say that the dark haired boy simply wanted to play and we can prefer to say that he is nice, which is the second possible answer to the question.	


*In CR condition, children listen to the sentences presented in the left column. In the MR condition, the sentences presented in the second column were added. In the MIR conditions, the sentences presented in the third column were added. For space issues, we excluded from this table all the statements that included the picture. The training can be find in Supplementary Material.*

The instructions for the metacognitive trainings differed with respect to the three conditions and took place after the story plot line was presented (see **Supplementary Material S02** for detailed descriptions of the different conditions). In the CR training, the child was told that, when faced with the same question, people tend to respond differently and was told which two different responses were possible (e.g., ‘you could say that he’s mean if you focus on what he did to the other boy’ vs. ‘you could say that he’s not mean if you focus on what he really wanted to do: throw the ball for the other boy to catch’). In the MR training, the control condition script was expanded to a total of eight instructions that focused on the child’s attention to the signs revealing the protagonist’s intentions, instructing the child not to forget to take these indices into account when making their judgment. Additional material was used during these instructions: a cloud-callout in order to emphasize the intention of the protagonist, and a yellow circle to highlight and facilitate the orientation of the child on the intention indices, which was placed over the cloud-callout. In the MIR training, the script and procedure for the MR training was further expanded, with an equal amount of new instructions (executive alerts; *n* = 8). These additional instructions aimed at focusing the child on the necessity to not only respond automatically to the fact that a boy was hurt, as this could make him forget to take into account other factors regarding the story (e.g., the protagonist’s intention). We also added further support material: a dashed gray cage, which was placed on the image of the boy getting hurt, in order to help the child understand that this is not the only element that should be taken into account when faced with the question. At the end of each metacognitive training, children were asked questions to verify that they understood the different elements of the story (see **Supplementary Material S02**. for a precise description of the questions asked). Answers were not taken into account for the final analysis but instead only served as a validation criterion for the training, including or excluding the child from the study.

### Procedure

**FIGURE 4:**
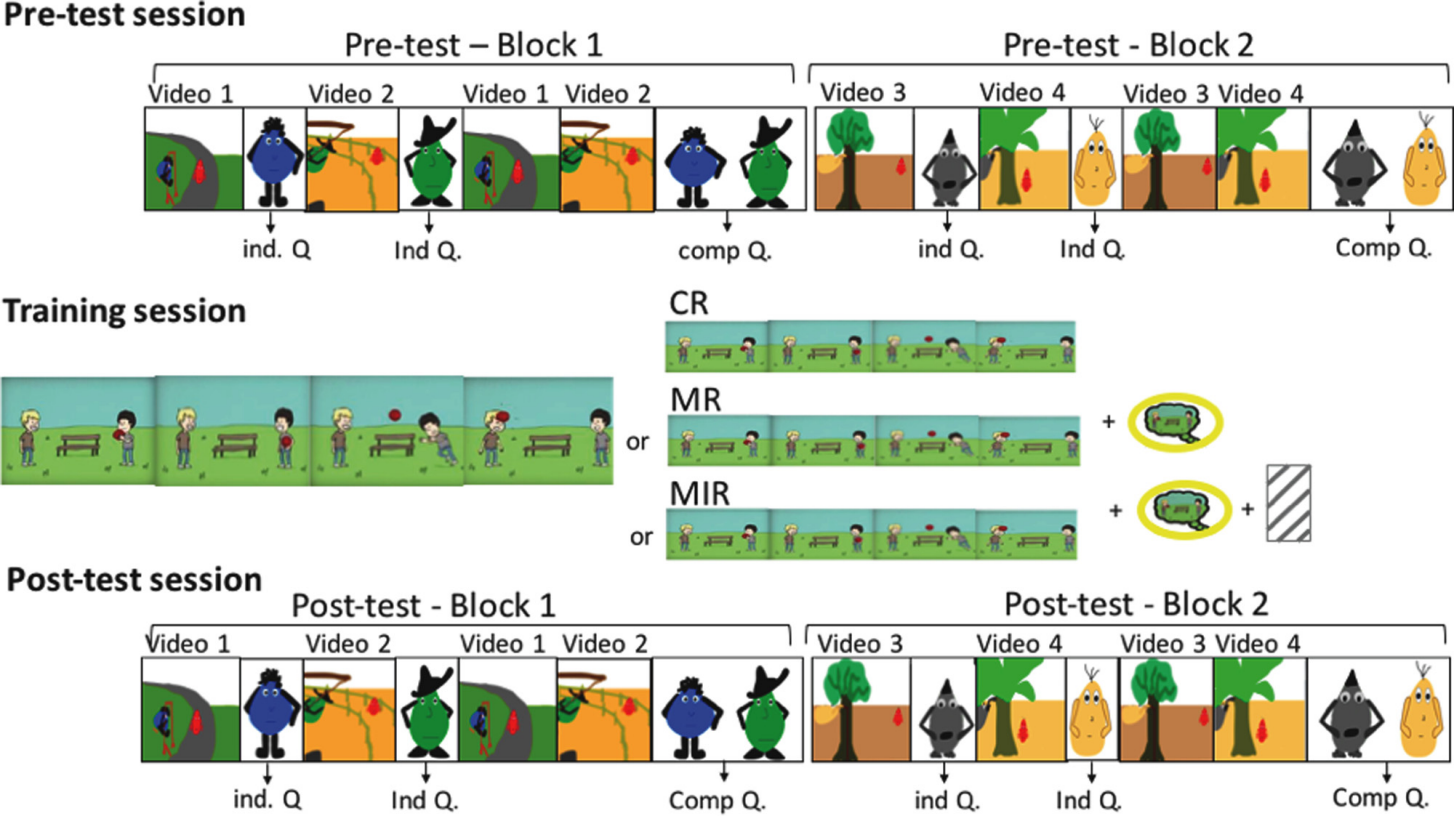
**Schematic representation of the procedure used with children.** In a pre-test session, children were presented with two blocks of videos designed to assess their sensitivity to the protagonists’ causal roles and to the protagonists’ intentions to harm. Each block contained a pair of videos. In a given block, each video was first presented, and followed by an individual questionnaire (Ind Q). The two videos were then presented again and followed by a questionnaire asking children to compare the two protagonists from a moral standpoint (Comp Q). During the metacognitive training, children were randomly assigned to one of the three conditions [control (CR), mentalising reinforcement (MR), or mentalising and inhibitory reinforcement (MIR)]. Finally, after the session with the metacognitive training, children underwent a post-training session identical to the pre-training session.

Children were recruited at “La cité des sciences” (Paris, France) with their parents during weekends. Children were first asked if they wanted to take part to an experiment consisting of watching some cartoons then answering questions about the. Then, parents of the children were explained the aims of the study and shown the video of one of the aggressive agents. If they thought this cartoon was appropriate to their children (which was the case for all the parents), they were given, the informed consent form to sign, and the experiment proceeded. The child took part in the experiment individually in a quiet room (see **Figure 4** for a schematic representation of the procedure followed). The child was first familiarized with the response buttons, with which they would later respond. This type of responding was choosen, since we hoped to collect children’s response time that are known to be highly relevant to measure the deployment of costly cognitive processes. However, we observed a really high variability across children: whereas some were really at ease with using the keys on the keyboard to answer, some children were not and only answered orally the questions that were asked, letting the experimenter press the buttons. As a result, the response times could not be a valid measure for subsequent analysis. This familiarization was followed by a pre-training session during which the child was presented with two different video pair contrasts (each child therefore saw four videos) across two blocks (see **Figure 4**). Each video was presented twice and followed by individual questions about the protagonists, allowing us to obtain children’s absolute evaluations of each protagonist (“Is he a good guy?,” “Is he a bad guy?,” “Do you want to play with him?,” and “Do you want to give him a gift?”). In order to answer, the children had to press the button (“yes,” “no,” “I don’t know” she/he was familiarize with before). Then both videos forming the contrast were presented again and were followed by a series of comparative questions, where the two protagonists were presented side by side: “Who is the bad guy?,” “Who is the good guy?,” “Who do you want to play with?,” and “Who do you want to give a gift to?.” To answer, the child had to press on computer keys that carry the colors of the characters. The experimenter asking the questions was blind to the videos presented to the child. If the child responded with “both” or “neither” on any of the comparative questions, the experimenter noted the answer by pressing a button on the keyboard; and if the child failed to respond, the experimenter noted the absence of response. A contrast pair of the Blue/Green protagonists was presented first (block 1) and secondly a contrast pair of the Gray/Yellow scenario (block 2). The color of the protagonist was partially counterbalanced (Mr. Blue vs. Mr. Green and Mr. Gray vs. Mr. Yellow), as well as the relative meanness of the protagonist in the first video of a contrast was counterbalanced (nicer vs. meaner), and the order of the protagonist’s role in the second contrast pair in regards to the first contrast pair (nicer/meaner – meaner/nicer vs. meaner/nicer – nicer/meaner).

Following the child’s answer to the last video, a second experimenter proceeded with the metacognitive training session. In all three conditions, we presented the images of the story plot-line one by one to the child. It should be noted that the hair color of the boy throwing the ball and the boy getting hurt was counterbalanced. After this presentation, we asked the child how he/she judged the boy who threw the ball. Subsequently smaller versions of all four images from the story timeline were put in front of the child, in chronological order, and we proceeded with the adequate training script. Once the script was finished, we asked the child what the possible answers to the initial question would be, and how could we justify them. Once the child could repeat the key elements, justifying the different responses, we considered the training as successful and continued on to the post-training session. If the child did not answer correctly, we repeated the resume of the training once again. Concerned with time limitations as well as biases that could occur due to an overexposure to the stimuli, we decided that if the child could not give correct explanations to the questions after the repetition of the resume, he was excluded from the analysis but nonetheless went on to finish the study.

The post-test session then followed and was identical to the pre-test presentation of the videos. Therefore any variation in the responses measured would be attributed to the effect of the metacognitive training.

## Results

### Scoring

In order to measure the extent to which children were able to distinguish the protagonists on the basis of the components manipulated in our paradigm using contrasts (the protagonist’s causal role and intention to harm), contrastive indexes were computed on the responses to the individual and comparative questionnaire as follows: for coding purposes the aggressive protagonist was considered the most ‘harmful,’ the coincidental protagonist the least ‘harmful,’ and the accidental protagonist intermediate. Responses in favor of the least harmful protagonist in a given contrast (the coincidental one for the causal contrast, the accidental one for the intentional contrast) were scored +1. Responses in the opposite direction were scored –1. A response “both” or “none” was scored as a zero. For each contrast (causal vs. intentional), a contrastive index was defined as the average of the 12 scores obtained for each individual and comparative questions in a given contrast (causal vs. intentional contrastive index). Each index ranged between +1 (preference for the less harmful protagonist) and –1 (preference for the more harmful protagonist). Note that individual questions allowed us to obtain participants’ absolute evaluations of each protagonist separately (i.e., whether a protagonist is evaluated positively or negatively on an absolute scale). We report all the analysis we conducted on absolute evaluations of protagonists in the **Supplementary Material S03**.

All the analyses were done using IBM SPSS Statistics 20. We report first the developmental effect obtained between children and adults’ moral judgments and then the metacognitive training effect.

### Developmental Effect

**FIGURE 5:**
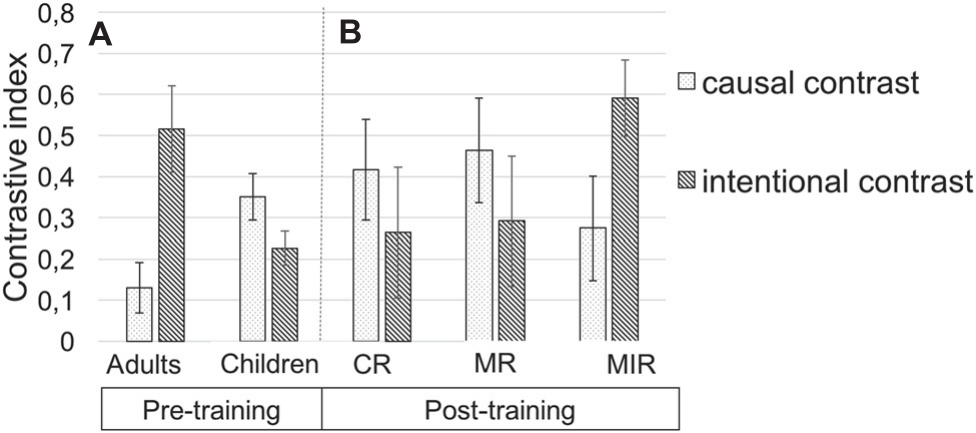
**(A)** Mean contrastive index obtained during the pre-test as a function of the age group (children vs. adults) and the contrast presented (causal vs. intentional contrast). **(B)** Mean contrastive index obtained for children during the post-test session as a function of the contrast presented and the type of metacognitive condition they previously underwent (control (CR) vs. mentalising (MR) vs. mentalising and inhibitory (MIR) reinforcements).

In order to explore the developmental effect between children and adults on the importance given to protagonists’ causal role and intentions to harm when generating moral judgment, we first conducted a general linear model (GLM) with contrastive indexes obtained for each contrast (contrast causal vs. intentional) during the pre-test as repeated measures, age of participants (adults vs. children), and counterbalancing factors (order of contrast, color of protagonists, order of protagonists’ presentation, sex of participants) as between subjects factors. Results revealed no effect of contrast [*F*(1,61) = 1.02, *p* < 0.1, 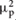 = 0.01] nor of age [*F*(1,61) < 1, *p* > 0.1, but did reveal a significant age by contrast interaction *F*(1,61) = 4.87, *p* < 0.05, 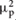 = 0.07]. GLMs conducted separately for each age group indicated that, in children, the contrastive index obtained for the causal index tended to be higher than the one obtained for the intentional contrast [*F*(1,56) = 2.92, *p* < 0.09, 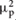 = 0.05] whereas in adults the contrastive index obtained for the intentional contrast was higher than the one obtained for the causal contrast [*F*(1,5) = 16.41, *p* = 0.01, 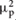 = 0.76]. Therefore, children tended to preferentially weight the protagonists’ causal role in their moral judgments while adults preferentially weighted the protagonists’ intentions in their moral judgment (**Figure 5A**). GLM conducted on each contrast separately indicate a main effect of age for the intentional contrast [*F*(1,87) = 4,99, *p* > 0.05, 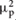 = 0.07] but not for the causal one [*F*(1,87) = 1, 67, *p* > 0.1, 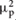 = 0.02]. Importantly, those developmental differences seem due to cases of accidental harm as children’s absolute evaluations of agents were comparable to adults’ ones for cases of aggression and coincidence but were more harsh for both cases of accidental harms (see **Supplementary Material S03**).

Despite this developmental difference regarding the respective weight given to the protagonists’ causal roles and their intentions to harm, it should be noted that the intercepts for the contrastive indexes obtained for the causal and the intentional contrasts were both significant for children (see **Table 2** for statistics). It indicates that children’s moral judgments were able to distinguish intentional harm from accidental harm as well as accidental harm from a mere coincidence. In brief, children are both sensitive to protagonists’ causal roles and intentions to harm. In adults, the intercept for the intentional contrast was significant and was only marginally significant for the causal contrast, indicating that adults integrated the protagonists’ intentions to harm in their moral judgment while they only had a tendency to integrate the protagonists’ causal roles. **Figure 5A** represents the mean contrastive index for each contrast presented (causal vs. intentional) and each age group (children vs. adults) during the pre-test.

**Table 2:** Significance of the intercept for each contrastive index obtained for each contrast (causal vs. intentional) and each group of participants (children vs. adults) during the pre-test, ^∗∗∗^*p* > 0.0001, ∼*p* < 0.1.

	Children	Adults
Causal contrast	*F*(1,56) = 35.5^∗∗∗^	*F*(1,5) = 4.92∼
Intentional contrast	*F*(1,56) = 28.97^∗∗∗^	*F*(1,5) = 209.34^∗∗∗^


### Metacognitive Training Effect

Before exploring the effect of our different metacognitive training, we first ensured that all groups of children were comparable on their moral judgment prior to the metacognitive training by conducting a GLM with the two indexes for each contrast (causal vs. intentional) obtained during the pre-test as repeated measures, the metacognitive training type (CR vs. MR vs. MIR) and all the counterbalanced factors as between subjects factors. Results showed no main effect of the group [*F*(2,27) < 1, *p* > 0.1] nor contrast by group interaction [*F*(2,27) = 1.58, *p* > 0.1], indicating that children’s moral judgments during the pre-test were similar across the three types of metacognitive trainings.

In order to explore the effect of our different metacognitive training types on children’s moral judgments, we conducted a GLM with the contrastive indexes for the two contrasts (causal vs. intentional) and the two times of testing (pre-test vs. post-test) as repeated measures, the metacognitive training type (CR vs. MR focus vs. MIR) as well as all the counterbalancing factors as between subjects factors. Results revealed no main effect of metacognitive training type [*F*(2,27) < 1, *p* > 0.1] but a main effect of the time of testing [*F*(1,27) = 4.17, *p* = 0.051, 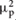 = 0.13), indicating that globally children’s contrastive indexes were higher after the metacognitive training than before the metacognitive training (mean contrastive index before the metacognitive training = 0.28, *SE* = 0.08, mean contrastive index after the metacognitive training = 0.38, *SE* = 0.10). This analysis did not reveal time of testing by contrast interaction [*F*(1,27) = 1.67, *p* > 0.1, 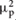 = 0.01] but revealed a time of testing by contrast by metacognitive training type interaction [*F*(2,27) = 3.55, *p* < 0.05, 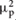 = 0.21]. This three-way interaction suggests that our different metacognitive trainings had distinctive effects on the way children weight the protagonists’ causal role and intention to harm before and after the metacognitive training. Therefore, to better understand the meaning of this interaction, we conducted for each metacognitive training separately, the same GLM as before except that the training type factor was removed from the analysis. **Figure 5B** represents the mean contrastive index for each contrast and each metacognitive training type obtained in children during the post-test session.

The analysis conducted on the CR and MR groups revealed no main effect of the contrast presented [CR group: *F*(1,8) = 1.56, *p* > 0.1, 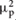 = 0.14; MR group: *F*(1,8) < 1, *p* > 0.1] nor of the time of testing [CR group: *F*(1,8) = 3.09, *p* > 0.1, 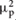 = 0.25; MR group: *F*(1,8) = 1.68, *p* > 0.1, 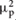 = 0.17] or contrast by time of testing interaction [CR group: *F*(1,8) < 1, *p* > 0.1; MR group *F*(1,8) = 1.70 *p* > 0.1, 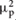 = 0.17]. This indicates that in these two groups, the metacognitive training procedures did not have any effect on children’s moral judgments.

By contrast, for children who underwent the MIR training, the analysis revealed no effect of contrast [*F*(1,8) = 1.05 *p* > 0.1, 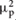 = 0.09] nor of time of testing [*F*(1,8) = 1.09 *p* > 0.1, 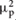 = 0.09] but a significant contrast by time of testing interaction [*F*(1,8) = 10.03, *p* = 0.01, 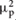 = 0.50], suggesting thus that the weight given to the protagonists’ causal roles over protagonists’ intentions did change in children who underwent the MIR training. In line with this, follow-up analyses revealed that, while children from this group gave equal weight to the protagonists’ causal role and the protagonists’ intention during the pre-test, [*F*(1,10) < 1, *p* > 0.1], they tended to preferentially weigh the protagonists’ intentions in their moral judgments during the post-test (i.e., the contrastive index is higher for the intentional contrast than for the causal contrast: [*F*(1,10) = 3.46, *p* = 0.09, 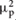 = 0.25]. This suggests, in line with the descriptive results, that the MIR training increases children’s use of information about intention while decreasing their reliance on information about the agent’s causal role. However, GLM conducted on each contrast taken separately with the time of testing as repeated measures and all of the counterbalanced factors as between subjects factor revealed a significant effect of the time of testing for the index obtained for the intentional contrast but failed to reach significance for the causal contrast [effect of the time of testing on the intentional contrast *F*(1,8) = 7.119, *p* < 0.05, 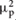 = 0.416; on the causal contrast: *F*(1,8) = 2.543, *p* > 0.1, 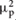 = 0.203].

In a final analysis, we wanted to check whether the pattern of judgments generated by children from different metacognitive training groups after the training differed from the adults’ pattern of judgment before the training. To do that, we compared children’s post-test evaluations of the different contrasts with adults’ pre-test evaluations of the very same contrasts. In children, who underwent the CR and the MR training, we found no main effect of age [CR: *F*(1,24) < 1, *p* > 0.1, MR: *F*(1,24) < 1, *p* > 0.1] or contrast [CR: *F*(1,24) = 1.27, *p* > 0.1, 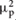 = 0.05; MR: *F*(1,24) < 1, *p* > 0.1] but still found the significant age by contrast interaction [CR: *F*(1,24) = 6.77, *p* < 0.05, 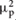 = 0.22; MR: *F*(1,24) = 6.05, *p* < 0.05, 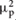 = 0.20] indicating that even after the metacognitive training children who underwent the control and the MR trainings continued to give different weights to protagonists’ causal role and intention to harm, as compared to adults. By contrast, in children who underwent the MIR training, analyses revealed only a main effect of contrast [*F*(1,24) = 5.77, *p* < 0.05, 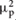 = 0.19] but no main effect of age [*F*(1,24) = 2.60, *p* < 0.1, 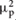 = 0.09] nor age by contrast interaction [*F*(1,24) = 2.60, *p* < 0.1, 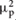 = 0.09], indicating that after the training, children who underwent the MIR metacognitive training did not weight the protagonists’ causal roles and their intentions to harm significantly differently as compared to adults.

## Discussion

The aim of the present study was to examine the cognitive processes involved in the development of a child’s capacity to integrate and prioritize a protagonist’s intention into his moral judgment. Specifically, we hypothesized that 5 to 8-year-old children’s inability to prioritize information about a protagonist’s intentions in their moral judgment was due to limitations in their ability to override the intuitive responses arising from the perception of a protagonist’s causal role in a victim’s suffering, particularly in the case where the protagonist’s harmful causal role conflicts with his/her innocent intentions in the judged moral situation (i.e., accidental harm). In order to test this hypothesis, we presented a series of videos and measured children’s sensitivity to the protagonist’s causal role and their intention to harm. Afterward, children underwent a metacognitive training aiming at increasing their focus and analysis of protagonists’ intentions, either with or without executive alarms aiming at reinforcing their ability to inhibit their intuitive reaction caused by the perception of the protagonist’s causal role. An additional training was included in order to control for any test–retest effects. Finally, in order to investigate the effect of our training on the child’s moral judgment, the child had to re-evaluate the same videos as seen prior to the metacognitive training.

Our results indicated that before the metacognitive training, while 5 to 8-year-old children were sensitive to both the protagonists’ causal roles and harmful intentions in their moral judgment, they were unable, contrary to adults, to generate a moral judgment dominated by the protagonist’s intentions. In addition, when exploring the impact of our metacognitive trainings, our results revealed that it had a selective effect in children who underwent a metacognitive training with MIR. Only with this type of metacognitive training children were able to change the relative weight given to the protagonist’s causal roles and intentions, in favor of the protagonist’s intentions.

These results first reproduce the developmental pattern of the ability to generate intent-based moral judgment that we described in the introduction. We especially confirm that, by the age of 5, children are sensitive to the protagonist’s intentions, while still being unable to integrate them as a priority in their moral judgment (Nelson-Le Gall, 1985; Shultz et al., 1988; Baird and Astington, 2004; Nobes et al., 2009; Cushman et al., 2013). Importantly, as demonstrated in the analyses, we conducted on participants’ absolute evaluations of protagonists (see **Supplementary Material S01**), from 5 to 8 years of age, children find it especially difficult to generate lenient judgments about protagonists committing accidental harm, thus confirming that the late development of intent-based moral judgment appears to be mostly due to a difficulty to integrate a protagonist’s innocent intention (Cushman et al., 2013).

Crucially, our study provides important insights into the processes responsible for this late emergence of mature intent-based moral judgment in regards to harmful situations, indicating that only children who were instructed to “not focus too much” on the consequences of the situation were able to show adult-like judgment after the metacognitive training. This result could not be taken as a test–retest effect nor as a side effect resulting from an over-exposure to situations of accidental harm since children who underwent the control training did not show any modification between the two times of testing. This result could neither be taken as the result of the MR in the metacognitive trainings since children who were (only) explicitly instructed to focus their attention on the intent/mental states did not progress more than children from the control group. In line with our hypothesis, it suggests 5 to 8-year-old children’s shortcomings to generate intent-based moral judgments are not due to their inability to focus on or to represent and correctly use the protagonist’s mental states, but their difficulty to override the intuitive evaluation based on the protagonist’s causal role.

Surprisingly, our results indicate that our inhibitory reinforcement training significantly impacts children’s ability to distinguish intentional harm from accidental harm, but not their ability to distinguish accidental harm from mere coincidences. Since our training was designed to increase children’s use of information about intentions and decrease their reliance on information about the action’s outcomes, we may have expected our training to have an opposite effect on the causal and intentional contrasts. This absence of effect on the evaluation of the agent’s causal role does not prevent us to claim that the ability to generate intent-based moral judgment relies on the ability to override its affective reaction to the perceived accidental harm. Indeed, our MIR training and the effect we obtained on the intentional contrast were sufficient to induce a shift from a tendency to favor agents’ causal roles to a tendency to favor agents’ intentions in children’s moral evaluations. In addition, as shown in the analyses of children’s absolute evaluations of agents (**Supplementary Material S03**), our MIR training strongly modifies children’s ability to integrate information about innocent intentions in both the context of the causal and the intentional contrast. An interesting potential explanation to the observed findings could be that the impact of our training on the evaluation of the agent’s causal role had been hidden by children’s more lenient evaluation of coincidence conditions after the training compared to before, making the differentiation between accidental and coincidence similar between the two times of the testing (for evidence in favor of this hypothesis, see **Supplementary Material S03**).

The present findings are congruent with recent models arising from cognitive neuroscience according to which an individual’s ability to make a moral judgment would depend on a dual-process system of evaluation (Cushman, 2008; Greene, 2009). Such processes occasionally conflict when what would be the most relevant response arises from a non-automatic processes and conflicts with an intuitive/automatic response that needs to be overridden, which requires cognitive control resources, (Greene, 2009). The validity of this type of model has already been demonstrated in the context of the now famous high conflict moral dilemmas (Greene, 2009), but also in adults’ evaluations of accidental harm (Young et al., 2007; Buon et al., 2013b, see also Treadway et al., 2014). The present study is the first to explore experimentally such an architecture in the *development* of moral competencies. By showing that executive alerts are critical to allow school-aged children to generate intent-based moral judgments, our results suggest that a dual processes model accounts for the protracted development of morality and that inhibitory control may be critical to provide mature moral judgment.

Our results are in line with a recent study that showed interesting neurodevelopmental changes in structures typically involved in affective saliency (i.e., amygdala and insula); responses in these areas decreased with age. Conversely, activity in regions of the medial and ventral prefrontal cortex, which are reciprocally connected with the amygdala and thought to be involved in resolving conflict, decision-making and evaluation, increased with age (Decety et al., 2011). This suggests thus that processing moral situations is more dependent upon basic affective responses in younger participants than in older participants. Finally, our findings are also in line with Richardson et al. (2012) who proposed that young children tend to base their judgments on an experience system, a system that encourages a focus on the outcome while older children would be more and more able to use a representational system, thanks to their growing executive functions that would permit to override the experience system. Whether the “cause-based heuristic” (that young children rely on and that the older ones seem to override) would depend on the child’s personal experience – as proposed by Richardson and collaborators – with intentional or accidental harmful actions would deserve further considerations in future research.

On the contrary, at first sight, this result could appear counter-intuitive given the recent studies which showed that ToM abilities were highly related to adults (Young et al., 2007; Young and Saxe, 2009; Buon et al., 2013a; Moran, 2013) and children’s abilities to generate intent-based moral judgment (Baird and Astington, 2004; Killen et al., 2011; Fu et al., 2014). However, as in the field of logical reasoning, demonstrating that inhibition is critical for solving a certain type of logical problem does not mean that logical reasoning is unnecessary (Houdé et al., 2000). Instead, it means that logical abilities are not sufficient to solve this type of problems. Similarly, we think that ToM abilities are necessary but not sufficient to acquire moral maturity: if the participants were unable to correctly reason about others’ mental states correctly, we think that our inhibitory-control training would be useless in helping them to generate intent-based moral judgments.

Related to this issue, it is important to recall that ToM capacities have been proven to be costly in executive resources and especially in inhibitory control resources (Carlson et al., 2004; Apperly et al., 2006). One may thus argue that our executive alert did not have an impact on the children’s ability to override the intuitive response arising from the perception of the protagonist’s causal role, but would permit children (and adults) to engage in, (or to sustain) the costly ToM reasoning that would be required to properly interpret the protagonist’s mental state. According to a first scenario, individuals would need to inhibit their intuitive reactions in order to engage themselves in ToM reasoning required to detect the protagonist’s intention. Consequently, inhibition would be required (and thus precede) to activate any relevant (ToM) reasoning. According to a second scenario, executive resources would help sustain the inferential process needed to detect a protagonist’s innocent intentions. For instance, one may hypothesize that executive resources may be especially useful in order for individuals to inhibit their own particularly arousing perspective (“I knew that he was about to cause harm!”). Both scenarios would predict that without sufficient executive and inhibitory resources, children (or adults) would be unable to correctly interpret a protagonist who caused harm. In favor of this hypothesis, an interesting finding arose from Killen et al.’s (2011) experiment that showed that children who passed the classical FBT were slightly worse at inferring a protagonist’s false–belief whose action caused harm, than that of a protagonist who did not cause harm in a morally relevant FBT. However, in a recent study – using the same stimuli as the ones presented in the current study – adults had to perform a concurrent verbal shadowing task while evaluating different situations of harm, and the authors controlled so that the participants under cognitive load conditions were able to properly represent the protagonists’ intentions (Buon et al., 2013b). The results revealed that in cognitive load conditions, whereas adults were above chance at representing the agents’ intentions, they were unable to incorporate them in their moral judgments. This suggests that the effect of the concurrent task especially impacts the *integration* of information about intentions into the moral judgment and not their *detection*^6^. Further studies are required to disentangle these different hypothesis that are critical to understand the interactions between ToM capacities and inhibitory control resources in the development of moral judgment.

Even though the present study provides promising evidence for a dual model in the ability to generate intent-based moral judgment — and more specifically for the importance of inhibitory processes in such a moral development — there are some limitations that should be addressed in order to validate more comprehensively our conclusions. Firstly our training paradigm only allowed us to measure the effect of trainings in a short timeframe: immediately after the trainings took place. This informs us only on the short term changes in children’s moral evaluation. However, it must be noted that this study was not conducted to observe long term learning in the moral domain, but rather to provide explanations for the late developmental shift from outcome based to intent based moral judgment. Secondly, our training paradigm was task specific since both the training and the test situations were about cases of accidental harm and therefore isomorphic tasks, which prevents us to test for a far transfer effect that our paradigm might produce on children’s evaluations. Importantly, the type of metacognitive training with inhibitory components that we used does not aim to increase children’s general inhibitory efficiency. Instead, the inclusion of emotional alerts (inhibitory component) together with instructions about the procedure to follow (mentalising component) aim to increase children’ *strategic* deployment of inhibition during the processing of moral situations, allowing them to override a particular response bias and activate a deeper reasoning about the presented situation. Using the same type of inhibitory training as ours in the field of logical reasoning, Houdé et al. (2000) compared individual participants performance on the post-test to their pre-test performance and showed an increase of activity within areas that have been typically involved in both inhibition (i.e., left-middle-frontal gyrus) and logical thinking (i.e., regions involved in inner speech, such as Broca’s area). Within the moral domain, Greene et al. (2004) showed an increased activity in areas that are typically involved in both inhibitory control and abstract reasoning in adults who were able to respond ‘rationally’ to the footbridge dilemma (compared to adults who were not). The evidence described above suggests that the inhibitory training we used may be efficient in both allowing the children to inhibit their intuitive causal evaluations, as well as activating deeper reasoning about the relevant factors involved in their decision. However, this suggestion raises the question of whether our training triggers a deeper reasoning about the agents’ mental states (ToM reasoning, see also discussion above) or a more generally increase in children’s ability to reason about non-intuitive components of the perceived situation. To answer this question, it would be highly relevant to investigate the generalization of our results by exploring whether a metacognitive training has an impact on children’s abilities to generate ‘rational’ responses in others types of moral dilemma that required to override a prepotent response, but in which ToM reasoning is critical or not.

Finally, another question that remains to answer in order to certify the critical role of inhibition in the developmental shift from outcome based to intent based moral judgment is whether a *pure* inhibitory training (e.g., a training involving inhibition of a non-moral or even non-social intuitive response without metacognitive components, see for instance Thorell et al., 2009; Diamond and Lee, 2011; Linzarini et al., 2015) would be efficient at enabling children to generate intent-based moral judgment. Since inter-tasks priming paradigms of this kind typically allow to investigate the degree of generalizability of inhibitory control (Linzarini et al., 2015), they would not only strengthen the validity of our conclusions but would also help us to deeply characterize the kind of inhibition (domain specific or general inhibition) that are involved in the processing of the ability to generate intent-based moral judgment.

## Conclusion

By showing that a metacognitive training that includes an inhibitory component induces an important shift in children’s moral abilities, our experiment suggests an important role of inhibitory control in the ability to generate intent-based moral judgment in school-age children. Our results extend findings indicated by Moutier (2000), which have shown that only a training procedure reinforcing the child’s capacity to inhibit an intuitive response bias would allow the child to give a logical answer. More broadly, our research clearly fits the hypothesis which posits that children are “inefficient inhibitors” (Houdé, 2000). This hypothesis has proven already to justify several “incompetence” of children, including logical reasoning (Moutier, 2000; Moutier and Houde, 2003) but also logico-mathematical reasoning (Houdé, 2000; Poirel et al., 2012; Borst et al., 2013) and logical categorization (Borst et al., 2012). Our findings suggest that children would also be inefficient inhibitors in the field of moral cognition. Beyond allowing us to shed a new light on an important theoretical challenge that represents the understanding of the cognitive architecture underlying moral development, our paradigm may also have an important long-term impact on the treatment of children with behavioral problems. Indeed, the use of others’ intentions in everyday social life interactions is critical for children’s harmonious social relationships (Arsenio et al., 2009). Notably, it has been shown that children who were more prone than their peers to the hostile attribution bias (i.e., the tendency to interpret all provocations as it seems guided by malevolent intentions) presented a high rate of reactive aggression (Orobio de Castro et al., 2002; Dodge et al., 2013). The understanding of the processes underlying this atypical information processing is crucial to improve the social life of these individuals by developing targeted new rehabilitation therapies.

## Author Contributions

MB and ED brought the theoretical background, SM had the idea of the experiment. KG, MB and SM designed the experiment, MB and KG tested the participants and analyzed the experiment. KG, MB, ED, and SM wrote the manuscript.

## Conflict of Interest Statement

The authors declare that the research was conducted in the absence of any commercial or financial relationships that could be construed as a potential conflict of interest.

## Footnotes

1 In the most common version of the FBT, children are shown a story involving two characters. For example, the child is shown two dolls, Sally and Anne, who have a basket and a box, respectively. Sally also has a marble, which she places in her basket, and then leaves the room. While she is out of the room, Anne takes the marble from the basket and puts it in the box. Sally returns, and the child is then asked where Sally will look for the marble. The child passes the task if she answers that Sally will look in the basket, where she put the marble; the child fails the task if she answers that Sally will look in the box, where the child knows the marble is hidden, even though Sally cannot know this, since she did not see it hidden there. To pass the task, the child must be able to understand that another’s mental representation of the situation is different from their own, and the child must be able to predict behavior based on that understanding.

2 In these studies, the term ‘metacognitive’ was used since the type of training the children underwent requested them to engage in high order thinking about the task and engaged the participants in a reflective process about the strategy they use to solve it.

3 In the classical, Wason task participants are presented with four cards. Each card has a letter on one side and a number on the other one. The participants are given a condition rule (respecting the form “if P, then Q”) – “if there is an ‘A’ on one side, then there is a ‘4’ on the other side.” The visible cards are ‘A,’ ‘D,’ ‘4’ and ‘7.’ Participants are asked to select all cards that must be turned over in order to verify if the given condition is true. A vast proportion of participants choose the cards ‘A’ and ‘4,’ which is not the logically correct response, which would be ‘A’ and ‘7’ (i.e., P and not-Q).

4 Any carry over effect of the pre-test was controlled by the control training, where the participants had equal exposure to the stimuli as in the other group.

5 When children were split into two age groups based on the median age (younger < 6.9 years-old, older > 6.9 years-old), we observed neither significant effect of age [*F*(1,70) < 1, *p* > 0.1] nor age by contrast interaction [*F*(1,70) = 2.76, *p* > 0.1,  = 0.3]. We thus decided to merge 5 to 8-year-olds children together. There was also no significant difference of age across the three groups [*F*(1,71) = 1.08, *p* > 0.1].

6 We did not insert questions about the protagonists’ intentions on purpose, considering that such information would be uninterpretable since participants may base their intentionality rating on their moral ratings as a *post hoc* justification, In addition, we did not want to insert such a training in our design since it could over-focus children on the protagonist’ intentions, biasing the potential impact of our different metacognitive trainings.
