# Supplementary material for: Priming Children’s Use of Intentions in Moral Judgement with Metacognitive Training
Source: Front Psychol. 2016 Mar 18;7:190. doi: 10.3389/fpsyg.2016.00190 (PMC4797364; doi:10.3389/fpsyg.2016.00190)
Supplement: Supplementary file 1 [file Presentation_1.PDF]

Supplementary material for the manuscript entitled “It’s not only mental states that count! A specific effect of executive training on children’s ability to generate intent-based moral judgment”

S01. Validation of the new set of stimuli on adults 2

S02. Detailed description of the training procedure 4

S03. Results obtained on the absolute valence indexes 10

S01. Validation of the new set of stimuli on adults

The stimuli we used in the pre and post-training sessions were already partially validated (see supplementary material in Buon et al., 2013, for validations of the cartoons with Mr. Blue and Mr. Green). A new set of cartoon involving Mr. Yellow and Mr. Grey as agents were created for the present experiment (a total of 6 cartoons were created : 3 conditions by agent). In order to validate the stimuli, we ran a pilot study on 8 healthy participants recruited at the Ecole Normale Supérieure.

Participants were presented with the 6 cartoons (Mr. Grey and Mr. Yellow each involved in Coincidence, Accident or Aggression conditions) in a random order. After each cartoon, participants were asked to rate their perception of the agent's causal (Does the agent have a causal role in what happens to Mr. Red?) and intentional role (Does the agent act on purpose?) using a 6 point scale (1 - not at all – to 6 – a lot).

Figure S01 presents the means of the causal and intentional evaluations for each condition perceived. Table 1 presents the means and standard deviation for each condition and agents separately. Results indicated a main effect of the condition for each evaluation (causal evaluation, $F(2,6)=67.02$, $p<.0001$; intentional evaluation, $F(2,6)=86.91, p<.0001$) , but no effect of the agent's color (effect of color for causal evaluation, $F(1,7)=1.06$, $p>.1$; intentional evaluation, $F(1,7)<1$, $p>.1$) or color by condition interaction ($F<1, p>.1$ for both evaluations). In order to examine the effect of condition for each evaluation, the grey and yellow agents were merged.

For the causal evaluations, follow-up t-tests revealed that participants considered the agents from the Aggression and the Accident condition to have the same causal role (difference Aggression/Accident: $t(7)=1.4$, $p>.1$) but considered the agents from the Aggression and Accident conditions to have a more causal role than the agent from the Coincidence condition (difference Aggression/Coincidence = $t(7)=11.81, p<.0001$; Difference Accident/Coincidence = $t(7)=5.38$, $p<.001$).

For the intentional evaluations, follow-up t-tests revealed that participants considered the

agent from the Aggression condition to have greater intention to harm than the agents from the Accident and Coincidence conditions (difference Aggression/Accident t(7)=10.93, p<.0001; difference Aggression/Coincidence : t(7)=7.80, p<.0001) but they did not distinguish the agents from the Accident and Coincidence conditions (difference Accident/Coincidence : t(7)=1,p>.1), indicating that they consider these two agents to have the same (absence of) intention to harm.

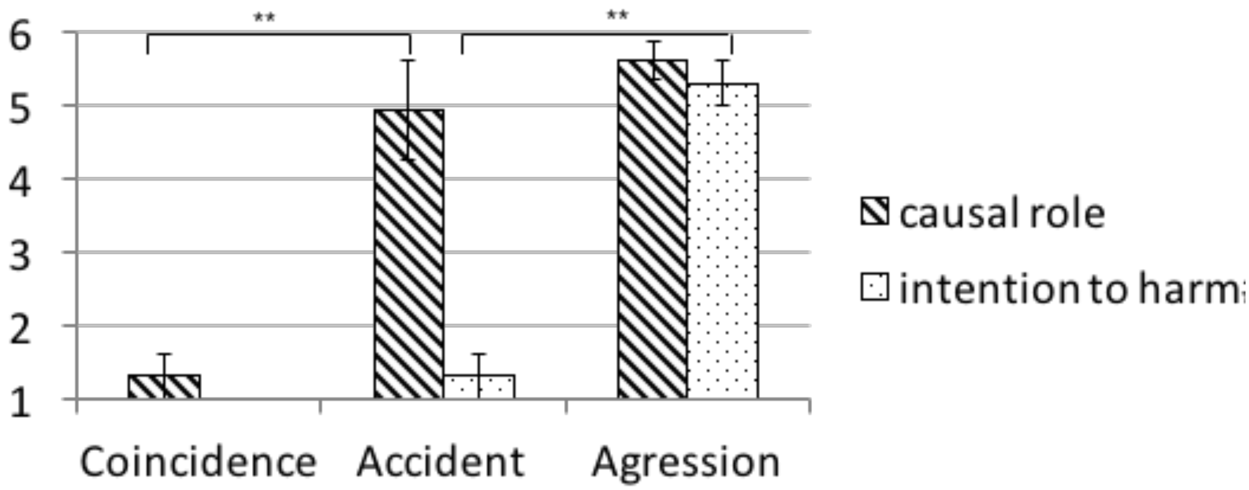

Figure S01: Mean causal and intentional evaluations for each condition perceived. On the scale, 1 means that the agent evaluated does not have any causal role/intention to harm. Only significant difference are displayed. ***, p<.0001

Table S01: Mean evaluation (and standard deviation) of the agents' causal role and intention to harm as a function of the condition and the color of the agent

	Agent's color	Aggression	Accident	Coincidence
Causal evaluation	Grey	5.62 (0.74)	4.87 (1.85)	1 (0)
	Yellow	5.62 (1.06)	5(1.92)	1.62 (1.76)
Intentionality evaluation	Grey	5.5 (0.92)	1 (0)	1(0)
	Yellow	5.125 (1.80)	1.625(1.76)	1(0)

S02. Detailed description of the training procedure

In the present experiment, the training procedure was adapted from the training protocols used in the domain of deductive reasoning (Moutier, 2000; Houde et al., 2001). The training session took place after the initial presentation of the videos and moral judgment assessment (Pre-training session). Note that different experimenters ran the pre-training and post-training sessions and the training session.

In order to introduce the training protocol, the second experimenter told the child the following script[1] :

> "Now I am going to tell you a short story about two boys who were playing with a ball. It is very important to listen carefully to what I am going to tell you. Try to remember and understand as much as possible from the story. Afterwards I am going to ask you to retell the story to me and respond to several questions."

Then, the experimenter laid down the first picture (see Figure 1) and said:

> "Here are two boys, a blond one and a dark haired one[2]. Can you show me which one is blond and which one is the dark haired?"

After the child successfully identified the boys, the experimenter continued to describe the first image:

> "The dark haired boy came to the park with a ball and asked the blond boy if he wanted to play with him, which the blond boy was happy to do".

Then the experimenter placed the second image over the first one and continued the story plot line:

---

[1] Originally done in French.

[2] Half of the children saw images with the dark haired boy throwing the ball and half of the children saw images with the blond boy throwing the ball.

"The dark haired boy then picked up the ball and prepared to throw the ball for the blond boy to catch".

The third image was then placed over the second one, accompanied by the next sentence:

"However, while throwing the ball the dark haired boy tripped and the ball was launched before time".

Finally the fourth image was placed over the previous ones and the experimenter concluded the story:

"The ball then fell on the head of the blond boy and hurt him".

The experimenter then turned the last image over so that no images were visible as not to bias the child's response and asked:

"In your opinion, is the dark haired boy, nice or is he mean?"

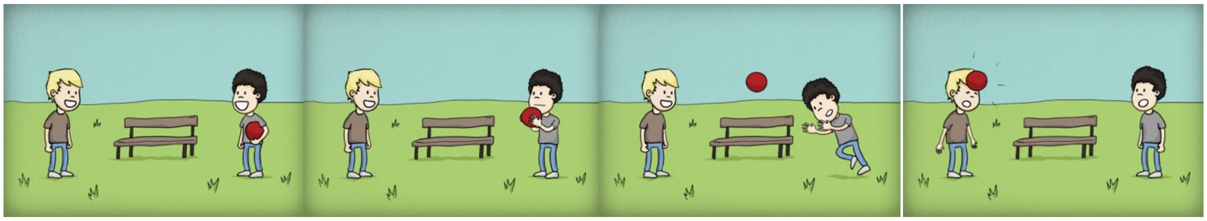

Image 1 Image 2 Image 3 Image 4

Figure S01 : **Baseline images used during the training session to illustrate the story plot-line presented to the child.** In the story, two boys are playing together. One of the boys is throwing the ball but accidentally trips and the ball falls on the other boy's head injuring the other boy.

The experimenter then took into account the child's response. He placed in front of the child the 4 images in a smaller format, and started the training itself using the script reported in table 2. There were 3 sets of statements: the control statements, the

mentalising statements and the executive alert statements. The control statements were told to all the children. The mentalising statements were additionally told to the children who underwent the mentalising and executive alert training. Finally, the executive alert statements were told only to the children who underwent the executive alert training.

Table S02: Statements told to the child as a function of the type of training.

<table>
<tr><th>Control training</th><th>Mentalising training</th><th>Executive Alert training</th></tr>
<tr><td colspan="3">There are people who respond like you to this question of whether the dark haired boy is nice or mean, saying that the dark haired boy is mean/nice (depending on the child’s answer). In fact, for this question there is actually more than one answer. Some people say that the dark haired boy is mean, while others say that he is nice. So I am going to place the same images, only in a smaller format in front of you and I suggest we look at all the different elements of the story in order to better understand how we can give these different responses.</td></tr>
<tr><td colspan="2"></td><td>Indeed, there is a trap here, which is to only look at certain elements of the story, for instance to only look at the blond boy getting hurt at the end of the story. The risk would be to forget other elements of the story that might help us respond to the question</td></tr>
<tr><td></td><td colspan="2">So, in order to respond to the question it is important</td></tr>
<tr><td colspan="2"></td><td>not to fall in to this trap, that is to say not to look at just one single element of the story and</td></tr>
<tr><td></td><td colspan="2">to look at all of the elements of the story, including what the brown haired boy wanted to do in the beginning of the story</td></tr>
<tr><td colspan="3">To better explain this, we will look at the different elements of the story together and at the different possible responses. To help you,</td></tr>
<tr><td colspan="2"></td><td>I will use this gray cage to hide the element that catches you in the trap</td></tr>
<tr><td></td><td colspan="2">with this yellow circle we will show the other element that we must not forget</td></tr>
<tr><td colspan="3">Let’s look at the different responses: if we only look at the blond boy being hurt at the end of the story after the dark haired boy threw the ball, we could have the need to say that the dark haired boy is mean.</td></tr>
<tr><td colspan="2"></td><td>However, in order not to fall into this trap, which consists of only looking at the blond boy being hurt after the ball fell on his head, I am going to place this gray cage over the boy getting hurt, in order to help us remember to look at other elements of the story which are also important in order to know if the dark haired boy is good or bad</td></tr>
</table>

If we go back to the beginning of the story and ask ourselves what the dark haired boy really wanted to do with the ball, another important element appears. (4) You can in this second picture that the boy took the ball in his hands and wanted to throw the ball so that the blond boy would catch it, and in the third picture you can see that he tripped and the ball flew in a direction he didn’t want to throw it. Since he tripped and the ball was launched in the bad direction, we can say that the dark haired boy didn’t want to hurt the bond boy so I will add this call out cloud with the image of what he actually wanted to do. Now, this yellow circle that I will put over it will help us remember not to forget what the dark haired boy actually wanted to do.

So, if we look at the fact that the dark haired boy did not actually want to hurt the blond boy, we can give a different response to the question of whether the dark haired boy is mean or nice, and we can say that he is in fact nice.

So, to summarize, when we see someone getting hurt, in order to say if the person who hurt him is nice or mean, we should try and look at all the elements of the story. So here we need :

First not to fall into the trap and the gray cage helps us do this. (15) We must not look at only the end of the story when the blond boy gets hurt, otherwise we could automatically respond saying that the dark haired boy is bad and we risk forgetting other elements from the beginning of the story which are important and can lead us want to a different response. Second, once we have avoided the trap, we can look at different elements of the story since it is also important.

To ask ourselves what the dark haired boy actually wanted to do at the beginning of the story , which the yellow circle helps us remember. Since the dark haired boy tripped and since the ball flew in an unwanted direction, we can say that the dark haired boy simply wanted to play and we can prefer to say that he is nice, which is the second possible answer to the question.

To validate the training, the child was asked a set of questions to ensure that he understood what the experimenter told him. Children who did not validate the training procedure were not included in the final analysis but did continue onto the post-training session since the experimenter presenting the pre-post sessions videos was blind to the training protocol and its validation.

The first question was: “Now tell me, what we can respond to the question “is the dark haired boy nice or is he mean?” For all the three training procedures the child had to give at least one of the possible answers.

The second question was “Why”, asking children to justify their answers since they generally did not do it automatically. Any explanation related to the different elements of the story was accepted since this question was only used to ensure that the child listened attentively to the story.

The third question was: “What is the element of the story that we must not forget when we want to respond to the question “Is the dark haired boy nice or is he mean?” ” If the child did not respond to the first formulation of the third question, the experimenter added the question: “What do we need to pay attention to?”. If the child responded by saying that we need to pay attention to the fact that the dark haired boy tripped, the experimenter asked for a justification to ensure that the child understood correctly.

The third question and its variations were used to validate both the mentalising training and the executive alert training. However responses to this question did not affect the validation of the control training.

For the mentalising and executive alert trainings, the question was validated if the child gave a response that clearly stated the intentions of the dark haired boy when throwing the ball. The child needed to give answers referring to mental states in order to validate the question. Simple responses only referring to facts stated in the story plot line or referring to the fact that the blond boy is hurt and sad did not reveal that a mentalising training took place (unless they were justified by elements referring to the intention of the dark haired boy).

Types of responses validating the question were: “He did not do it on purpose”, “He did not want to hurt the blond boy”, “The dark haired boy only wanted to play with the blond boy”, “The dark haired boy wanted to pass the ball to the blond boy”, “The dark haired boy did not want to throw the ball on the head of the blond boy”.

Types of responses that were not accepted as valid were: “The dark haired boy tripped”, “The ball is important”, “When the blond boy is hurt”, and all other responses not related to the story.

The final question asked was : “What is the element of the story that we should not focus on too much when we want to respond to the question?”.

A valid response to this question was needed to validate only the executive alert training, responses to this question being unnecessary in the other training groups.

In order to validate this fourth question, children needed to refer to the consequences of the actions. For example: “The blond boy being hurt”, “The ball falling on the head of the blond boy and him being sad”.

If the child could not respond correctly to one of the questions required for the validation of the training, the experimenter would repeat the summary (last paragraph) of the training once (and only once). If the child still did not give responses to questions needed for the validation, then the training was considered invalid and the child’s results were excluded from the study.

S03. Results obtained on the absolute valence indexes

As described in the main manuscript, individual questionnaires allow us to obtain absolute evaluations of agents which is of great interest for understanding whether the developmental and training effects we obtained rely on developmental changes regarding the accidental agents and/or the others.

Scoring

For the purpose of analysing participants' absolute evaluations, for each agent, we computed an Absolute Valence Index (AVI) based on the responses only to the individual questionnaires: first, each answer in favour of the agent (i.e., responses "Yes" to the questions "Is he a good guy?", "Do you want to play with him?" and "Do you want to give him a gift?", and "No" to the question "Is he a bad guy") was scored as +1, and each answer in disfavour of the agent was scored as -1. The AVI was defined as the average of the four scores, thereby ranging between +1 (positive evaluation)[3] and -1 (negative evaluation). A score above 0, below 0, or not significantly different from 0 means, respectively, that the agent is evaluated positively, negatively, or neutrally. In both cases, if the child does not answer the question of the experimenter, no score was assigned to the answer (NA).

Note that given the contrastive structure of our design, the agent from the accident condition was always considered in the context of its contrast: accident presented in the causal contrast (accident/CC) or accident presented in the intentional contrast (accident/CI). Hence a total of four AVIs was used for our analysis (AVIs for the coincidence, accident/CC, accident/CI, coincidence). As in the main manuscript, we will first describe the developmental effect obtained on the AVI, then the training effect.

3

Developmental effect on the evaluation of different conditions – absolute index

In order to explore the developmental effect on the absolute moral evaluation of each agent, we conducted a general linear model (GLM) with the AVIs obtained for each condition during the pre-test as repeated measures, age of participants (adults vs. children) and counterbalancing factors (order of contrast, color of agents, order of agents' presentation, sex of participants) as between subjects factors. Results revealed a main effect of age ($F(1,61)= 7.01$, $p<.01$, $\mu p^2 = .1$) and a main effect of condition ($F(3, 59) = 30,90$, $p<.0001$, $\mu p^2 = .34$),characterized by an age by contrast interaction ($F(3,59) = 5,64$, $p<.001$, $\mu p^2 = .08$). Post hoc analyses revealed that, for both adults and children, the AVI for the Coincidence condition was more positive than the one obtained for the Accident/CC condition (children: $F(1,56) = 25.03$, $p<.0001$, $\mu p^2 =.31$; adults : $F(1,5) = 13.97$, $p<.01$, $\mu p^2 =.73$). The AVI for the Accident/CI condition was also more positive than the one obtained for the Aggression condition (children: $F(1,56) = 7.97$, $p<.01$, $pp^2 =.12$; adults : $F(1,5) = 60.86$, $p<.001$, $\mu p^2 =.92$). However, age differences appeared on the AVIs for the two Accident conditions (CI and CC), children's AVIs for the accident/CC and accident/CI being more negative compared to adults (age difference, Accident/CC: $F(1,87) = 6.24$, $p<.05$, $\mu p^2 =.08$, Accident/CI: $F(1,87)=10.10$, $p<.0001$, $\mu p^2 =.20$). By contrast, no difference appeared on the AVIs for the Aggression ($F(1,87)= 1.72$, $p>.1$, $\mu p^2 =.02$) and Coincidence conditions ($F(1,87)= 2.58$, $p>.1$, $\mu p^2 =.03$). Relatedly, examination of the intercepts for each AVI (see table 1) indicated that both children and adults' AVIs for the Coincidence and Aggression condition were significantly positive and negative, respectively. By contrast, whereas the AVIs for the Accident/CC and Accident/CI conditions were neutral and positive, respectively, in adults, they were both negative in

children. Figure 2a represents the mean AVIs obtained for each condition and age group during the pre-training session.

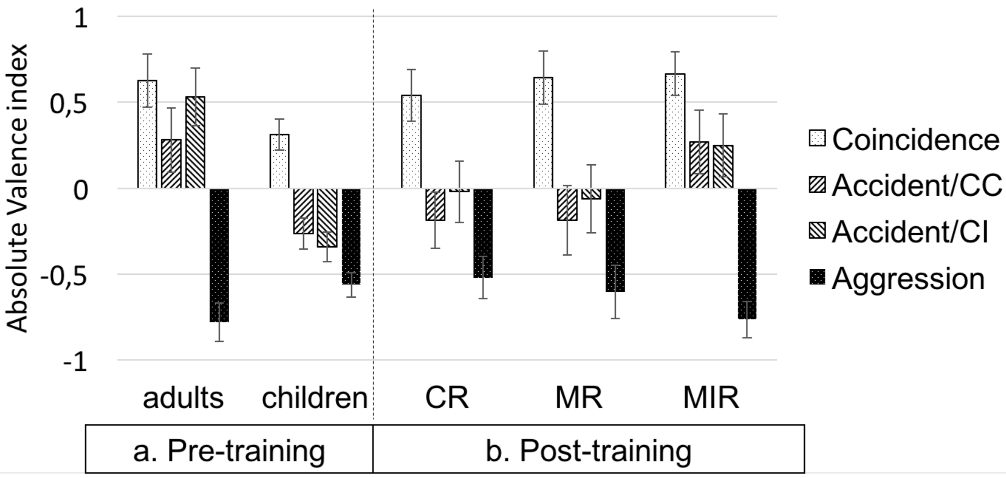

*Figure S03***:** a. Mean absolute valence index obtained during the pre-test as a function of the age group (children vs. adults) and the condition presented (coincidence vs. accident/CC vs. accident/CI vs. coincidence). A significant age by condition interaction was observed at $p<.001$. b. Mean absolute valence index obtained for children during the post-test session as a function of the contrast presented and the type training they previously underwent (control vs. mentalising vs. executive alert). A significant interaction of training group and time of testing was observed at $p<.05$.

Table S2: Examination of the significance for each AVI's intercept as a function of the condition perceived and the age group.

	Children	Adults
Coincidence	$F(1,64) = 18.79$, $p<.0001$, $\mu p^2 = .22$	$F(1,8) = 16.66$, $p<.01$, $\mu p^2 = .20$
Accident/CC	$F(1,64) = 7.43$, $p<.01$, $\mu p^2 = .10$	$F(1,8) = 2.07$, $p>.1$, $\mu p^2 = .20$
Accident/CI	$F(1,64) = 15.45$, $p<.0001$, $\mu p^2 = .19$	$F(1,8) = 8.75$, $p<.05$, $\mu p^2 = .52$
Aggression	$F(1,64) = 64.63$,	$F(1,8) = 56.81$, $p<.0001$, $\mu p^2 = .87$

NB: A significant intercept means that the AVI obtained is significantly above or below 0 (i.e., that the agent is evaluated positively or negatively, respectively)

Training effect on the absolute valence index

In order to study the effects of our training procedure on children's absolute evaluation of agents, we first ensured that children from the different training groups did not differ on their evaluations of the different agents before the training. Therefore, we first conducted a GLM with the AVIs obtained for each condition (Coincidence, Aggression, Accident/CC, and Accident/CI) as repeated measures, the training type (control, MR or MIR) and all the counterbalancing factors as independent factors. The results revealed a significant effect of the condition ($F(3,25)=28.26$, $p<.0001$, $\mu p^2=.51$) but no main effect of the training type ($F(2,27)<1$, $p<.1$) nor condition by training interaction ($F(6,52)=<1, p>.1$), indicating that the children in the 3 training groups did not differ in their evaluations of the different conditions before the training.

Figure 2b represents the mean AVIs for each condition and each training type obtained in children during the post-training session. In order to explore the effect of our training procedure on children's evaluations of the different conditions, we conducted a GLM with the AVIs obtained for each condition and the time of testing (pre vs. post training) as repeated measures, the training type and all the counterbalancing factors as independent factors. Results revealed no main effect of training type ($F(2,27) = 1.71$, $p<.1$, $\mu p^2=.02$) nor condition by type of training interaction ($F(6,52)<1$, $p>.1$). However, we found a main effect of condition ($F(3,25)=37.81$, $p<.0001$, $\mu p^2=.58$) and a main effect of the time of testing ($F(1,27)=56.66$, $p<.0001$, $\mu p^2=.67$) characterised by a time of testing by training

($F(2,27)=4.18$, $p<.05$, $\mu p^2=.23$), a condition by a time of testing ($F(3,25)=4.75$, $p<.01$, $\mu p^2=.15$) and a condition by time of testing by training type ($F(6,52)=2.19$,$p<.05$, $\mu p^2=.15$) interactions. These interactions suggest that our training procedure had an impact on children's evaluations of the different conditions, an impact that differed as a function of the type of training underwent by children. In order to understand the meaning of these different interactions, we conducted separate analyses for each training type. For each training type, we conducted a GLM with the AVIs obtained for the different conditions and the different times of testing (pre vs. post. training) as repeated measures, and all the counterbalancing factors as independent factors. Then, to characterize the effect of testing for each condition, we conducted a GLM with the time of testing as repeated measures and all the counterbalancing factors as independent measures for each condition separately.

For children who underwent the control training, results revealed a main effect of the condition ($F(3,7)=8.02$, $p<.001$, $\mu p^2=.47$), a main effect of the time of testing ($F(1,9)=9.81$, $p<.01$, $\mu p^2=.52$) but no condition by time of testing interaction ($F(3,7)=1.41$, $p>.1$, $\mu p^2=$ 13). Analyses conducted on the AVIs obtained for each condition separately (see table 2) revealed no significant effect of the time of testing on the AVIs obtained for the Accident/CC and Aggression conditions. However, we found that the AVIs for the Coincidence and Accident/CI condition showed a trend towards significance and were significantly higher, respectively, after the training compared to before.

For children who underwent the mentalising training, results also revealed a main effect of condition ($F(3,6)=33.53$, $p<.0001$, $\mu p^2=.81$), a main effect of the time of testing ($F(1.8)=21.78$, $p<.01$, $\mu pp^2=.73$ but no condition by time of testing interaction ($F(3,6)=2.05$,

$p>.1$, $\mu pp^2=.20$). Analyses conducted on the AVIs for each condition separately revealed no significant change on the evaluations of the Accident/CC and Aggression conditions. However, we found that the AVIs obtained for the Accident/CI condition showed a trend toward being higher after the training procedure while the one obtained for the Coincidence condition was significantly higher.

For children who underwent the MIR training, results revealed a main effect of condition ($F(3, 8)=14.96$, $p<.0001$, $\mu p^2=.59$), a main effect of the time of testing ($F(1,10)=30.36$, $p<.0001$, $\mu pp^2=.75$), and a condition by time of testing interaction ($F(3,8)=7.74$, $p<.001$, $\mu p^2=.43$). Analyses conducted on the AVIs for each condition separately revealed that the AVIs were significantly higher after the training for the Coincidence, Accident/CI and the Accident/CC condition but no change was observed for the Aggression condition.

In sum, we found that all the children – irrespective of the training they underwent - were significantly (or tended to be) less severe with the agents from the Coincidence and the Accident/CI conditions after the training compared to before. These effects seem to reflect the main effect of time of testing that we observed on the contrastive indexes (i.e., they were significantly higher after the training when compared to before the training). Given the comparative nature of our paradigms, this test-retest effect could reflect the fact that during the post-training session, children have understood that there was always a "meaner" and "nicer" agent in each contrast presented, which became more differentiated after the training. We also found a specific effect of the time of testing on children who underwent the MIR training, as these children would become less severe in their evaluation of the accident/CC condition after the training. This effect is opposite to the test-retest effect and clearly reflects the contrast by time of testing interaction we observed

on the contrastive indexes, according to which children who underwent the executive alert training changed the importance they ascribed to the agents’ causal role and intention to harm during the post-training session in comparison to the pre-training session.

Table S03: Examination of the effect of our different training procedure of children’s evaluation of the different agents.

	Control	Mentalising	Executive Alert
Coincidence	$F(1,9)=3.61, p=.09, \mu p^2=.28$	$F(1,8)=30, p=.001, \mu p^2=.78$	$F(1,9)=6.16, p<.05, \mu p^2=.38$
Accident/CC	$F(1,9)<1, p>.1$	$F(1,9)<1, p>.1$	$F(1,10)=16.50, p<.01, \mu p^2=.40$
Accident/CI	$F(1,9)= 8.26, p<.05, \mu p^2=.47$	$F(1,8)=4.26, p=.07, \mu p^2=.34$	$F(1,10)=15.71, p<.01, \mu p^2=.61$
Aggression	$F(1,9)<1, p>.1$	$F(1,9)<1, p>.1$	$F(1,10)=2.18, p=1.70, \mu p^2=.17$

.
